# Estimation of the ideal correction of lumbar lordosis to prevent reoperation for symptomatic adjacent segment disease after lumbar fusion in older people

**DOI:** 10.1186/s12891-020-03463-3

**Published:** 2020-07-03

**Authors:** Shan-Jin Wang, Shu-Bao Zhang, Yu-Yang Yi, Hao-Wei Xu, De-Sheng Wu

**Affiliations:** Department of Spinal Surgery, Shanghai East Hospital, Tongji University School of Medicine, 150# Jimo RD, Pudong New Area, Shanghai, 200120 China

**Keywords:** Lumbar fusion, Symptomatic adjacent segment disease, Disc degeneration, Correction of lumbar lordosis, Risk factor

## Abstract

**Background:**

Symptomatic adjacent segment disease (ASDis) is a major complication following spinal fusion. Sagittal spinopelvic imbalance may contribute to the development of ASDis. However, the exact ideal correction of lumbar lordosis (LL) is unknown for different ages of people to prevent ASDis. The purpose of this study was to estimate the ideal correction of LL required to prevent symptomatic ASDis requiring revision surgery in patients of various ages, and to determine the radiographic risk factors for ASDis.

**Methods:**

468 patients who underwent lumbar fusion between January 2014 and December 2016, were enrolled in the present study. The patients were classified into the ASDis and N-ASD group. These two matched groups were compared regarding surgery-related factors and radiographic features. Multivariate logistic regression analysis was used to evaluate the risk factors for ASDis.

**Results:**

Sixty-two patients (13.25%) underwent reoperation for ASDis during a mean follow-up duration of 38.07 months. Receiver operating characteristic curve analysis showed that the postoperative LL - preoperative LL (△LL) cutoff value was 11.7°for the development of ASDis. Logistic regression analysis revealed that the risk factors for symptomatic ASDis were a smaller LL angle, △LL > 12°, and PI-LL > 10° (*p* <  0.05). For patients > 60 years, the incidence of ASDis was higher in patients with a LL correction of ≥10° and a lumbar-pelvic mismatch (PI-LL) of > 20°.

**Conclusions:**

The significant predictors of the occurrence of ASDis were a smaller LL angle, △LL > 12°, and PI-LL > 10°. However, in patients older than 60 years, the incidence of ASDis after lumbar fusion was higher in those with a LL correction of ≥10° and PI-LL of > 20°. More attention should be paid to patient age and the angle of correction of LL before lumbar fusion.

## Background

With the rapid development of spinal surgery techniques, spinal fusion has become an established and common treatment for lumbar degenerative disease (LDD). However, long-term studies have found that adjacent segment degeneration (ASD) is common after lumbar fusion, with radiological ASD seen in 36–100% of patients and symptomatic ASD seen in 0–27.5% of patients [1–3]. There is no definitive gold standard for the diagnosis of ASD, but the most common manifestation of ASD is intervertebral disc degeneration at adjacent segments. ASD also includes segment instability, facet joint hyperplasia, and spinal canal stenosis. LDD frequently causes low back pain (LBP), and the economic cost of diagnosing and treating LBP in the United States is estimated at about $90 billion per year [4]. There is a high incidence of reoperation for ASD after spinal fusion, which may bring a great economic burden.

Although many studies have investigated the pathomechanism of ASD after spinal fusion, the conclusions are still controversial. Lumbar fusion may increase the stress on the nonoperative adjacent segments, leading to ASD in long-term follow-up [5]. However, ASD may be caused by natural degeneration of the spine. In addition, patient factors such as older age, obesity, pre-existing ASD, facet degeneration, and lumbar amyotrophy may contribute to the development of ASD [6]. Recent studies have shown that the sagittal spinopelvic balance significantly affects the clinical therapy of patients with LBP [7, 8]. LDD are often associated with spinopelvic imbalance. A decrease in lumbar lordosis (LL) is related to LBP, and overcorrection of LL is an effective therapeutic modality to maintain optimal sagittal alignment in patients with degenerative lumbar kyphosis [9, 10]. Patients with a pelvic incidence-LL (PI-LL) mismatch (PI-LL ≥ 10°) are 10 times more likely to develop ASD than patients with a PI-LL of < 10° [11]. However, sagittal spinopelvic alignment often changes with age, as older adult patients compensate for LL loss by allowing the trunk to pitch forward [12]. Thus, excessive pursue ideal alignment objectives are counterproductive for older adults.

The present study aimed to evaluate whether the incidence of reoperation for ASD after posterior vertebral fusion was associated with the age at the time of surgery and various pelvic parameters. Furthermore, we aimed to estimate the ideal correction of LL to prevent symptomatic ASD, optimize the clinical treatment plan, and improve the treatment effect.

## Methods

### Study population

The present study received ethical approval from the Ethics Committee Board of the participating hospital. We reviewed 667 patients who underwent posterolateral fusion (PLF) or posterior lumbar interbody fusion (PLIF) for LDD between January 2014 and December 2016. All patients under general anesthesia, classic posterior lumbar fixation fusion procedure was performed. Posterior lumbar pedicle screw internal fixation, laminectomy and nerve decompression. During the operation, more attention to avoid injury of the adjacent facet joints. Intervertebral, intertransverse and posterolateral bone graft were used for fusion. All patients used the same surgical implant instruments.

The inclusion criteria for patients in the reoperation group were: (1) symptomatic ASD disease diagnosed in patients with LBP, intermittent claudication, radiculopathy, or lower extremity muscle strength weakness that matched the radiographic ASD features (lumbar spinal stenosis or lumbar spondylolisthesis, disc degeneration, facet joint osteoarthritis); (2) complete imaging data; (3) primary lumbar fusion level between L1 and L5 for LDD. The exclusion criteria were: (1) lumbar trauma, infection, tumor, or congenital deformity; (2) sagittal vertical axis (SVA) > 5 cm or degenerative lumbar scoliosis > 20°; (3) refusal to participate in this study.

Four-hundred-and-sixty-eight patients were enrolled. The mean follow-up duration was 38.07 months. Of these 468 patients, 74 (15.81%) developed asymptomatic ASD, 62 (13.25%) required reoperation for symptomatic ASD after failure of conservative therapy including medication and/or physical treatment (ASDis group). These 62 patients were matched in a 1:1 ratio by sex, age, body mass index (BMI), follow-up duration, and other factors with enrolled patients who underwent posterior lumbar fusion but did not develop ASD (N-ASD group). The groups were created with similar distributions of matched variables to minimize selection bias before the radiographic and magnetic resonance imaging (MRI) measurements.

### Data collection

**Fig. 1:**
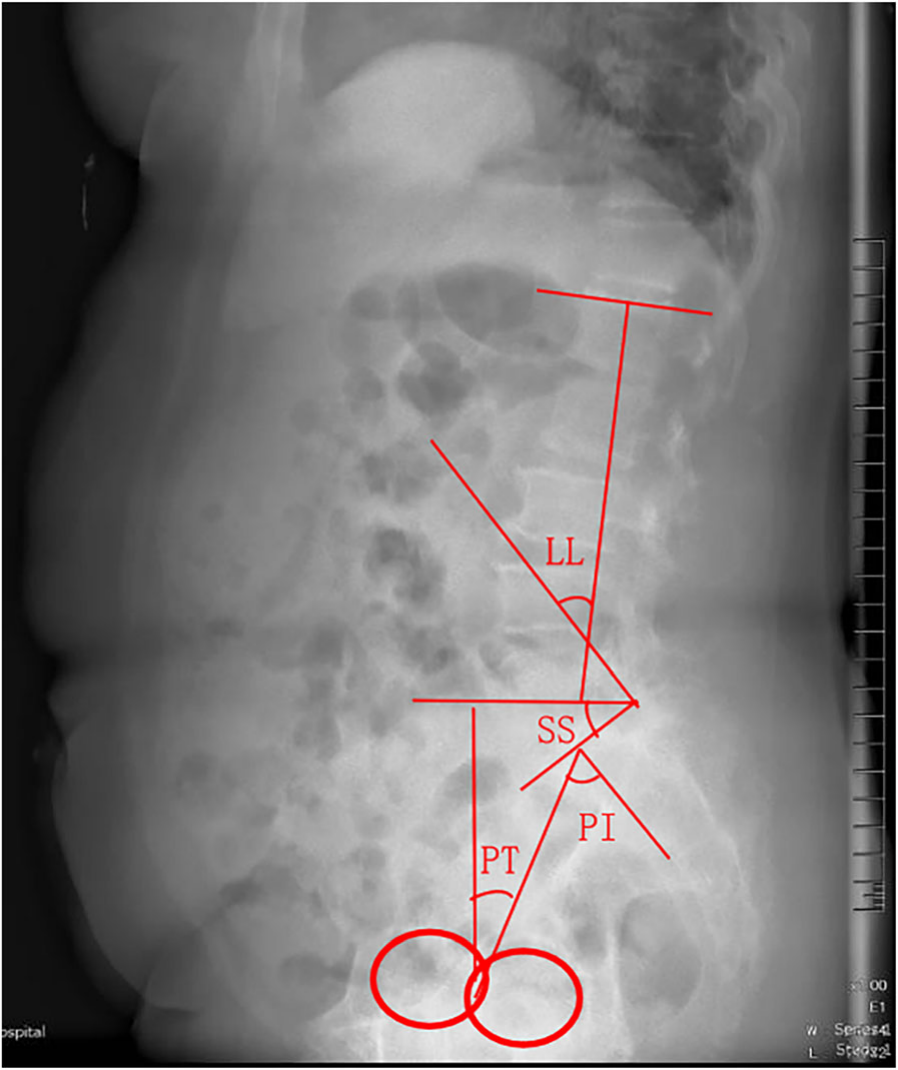
Methods for measuring the pelvic parameters. Lumbar lordosis (LL): angle between the superior endplate line of L1 and S1. Pelvic incidence (PI): angle between the perpendicular to the sacral plate at its midpoint and the line connecting this point to the middle axis of the femoral heads. Sacral slope (SS): angle between the superior plate of S1 and a horizontal line. Pelvic tilt (PT) = PI-SS

Plain radiography and MRI showed no degeneration or instability in the adjacent segments before the primary operation. Standing lumbar spine lateral radiographs (including the bilateral femoral heads) were taken for all patients. 1 week before and after surgery, pre- and postoperative sagittal spinopelvic parameters were measured to determine the SVA, LL, PI, sacral slope, pelvic tilt (PT), and PI-LL (Fig. 1); △LL was calculated as absolute value of the difference between the postoperative LL and the preoperative LL (△LL = |Postoperative LL - Preoperative LL|). On MRI, all patients in the ASD group had a Pfirrmann [13] disc degeneration grade of ≥ III at the adjacent segment and spinal canal stenosis (defined as a spinal canal midsagittal diameter of < 12 mm) [14]. Interviews and questionnaires were used to determine patient age, sex, BMI, smoking status, presence of hypertension, presence of diabetes mellitus, and drinking status.

### Statistics

Data of the abovementioned sagittal parameters were statistically analyzed using SPSS 25 (SPSS Inc., Chicago, IL, USA). We selected the controls using propensity score-matched (PSM) analyses, the 1:1 nearest neighbor technique with a small caliper of 0.2 to ensure better balance. Values were described as the mean ± standard deviation. If the data were normally distributed, the independent sample t-test was adopted to compare the ASD group and the N-ASD group. The Mann-Whitney U test was used to analyze differences in pelvic sagittal parameters that were non-normally distributed. Count data were analyzed using the chi-square test. The threshold value of positive results were obtained by receiver operating characteristic curve analysis and area under the curve quantitative analysis. After univariate analyzed, variables with statistically significant differences (*p* <  0.05) were incorporated into the multivariate logistic regression model. Multivariate logistic regression analysis was used to identify the risk factors for ASD. *p* <  0.05 was considered statistically significant.

## Results

### General situation

Before the initial surgery, there were 30 patients (48.4%) with degenerative spondylolisthesis, 12 (19.4%) with degenerative disc herniation, and 20 (32.3%) with foraminal stenosis in ASD groups (Table 1). During the 3-years follow-up, 44 patients (70.97%) had ASD at the cranial adjacent segment, while 18 (29.03%) had ASD at the caudal adjacent segment. The ASD group included 37 females and 25 males with an average age of 65.8 years. In the ASD group, there were 24 cases of PLIF and 38 cases of PLF; in the N-ASD group, there were 29 cases of PLIF and 33 cases of PLF. In the ASD group, the surgery level was L4-L5 in 26 patients, L3-L5 in 23, and L2-L5 in 13; in the N-ASD group, the surgery level was L4-L5 in 21, L3-L5 in 20, and L2-L5 in 21. There were no significant differences between the two groups regarding baseline data such as sex, age, BMI, smoking status, basic diseases, follow-up duration, number of cages, and surgical level (Table 1, *p* > 0.05).

**Table 1:** Comparison of patient characteristics between ASDis group and N-ASD group

Characteristic	ASDis (*n* = 62)	N-ASD (n = 62)	χ^2^/t	*p*
Age, year	61.65 ± 8.43	61.39 ± 8.14	0.17	0.863
BMI, kg/m ^2^	24.75 ± 3.27	24.73 ± 3.20	0.03	0.975
Sex (M/F)	25/37	25/37	0	1
Diabetes (Y)	19 (30.6%)	20 (32.3%)	0.04	0.847
Hypertension (Y)	34 (54.8%)	25 (40.3%)	2.62	0.106
Smoking (Y)	8 (12.9%)	9 (14.5%)	0.07	0.794
Drinking (Y)	8 (12.9%)	10 (16.1%)	0.26	0.610
Lumbar BMD (T scores)	−1.66 ± 1.33	−1.34 ± 1.40	−1.29	0.2
Follow-up (months)	37.98 ± 6.93	38.35 ± 8.04	−0.275	0.784
Disease				
DS	30 (48.4%)	22 (35.5%)	3.23	0.199
FS	20 (32.3%)	20 (32.3%)		
DH	12 (19.4%)	20 (32.3%)		
Fusion method				
PLF	38 (61.3%)	33 (53.2%)	0.824	0.364
PLIF	24 (38.7%)	29 (46.8%)		
Segments fused				
≤ 2 segments	29 (46.8%)	27 (43.5%)	0.13	0.718
> 2 segments	33 (53.2%)	35 (56.5%)		

Values are presented in mean ± standard error (SE) or percentages

*Y* Yes, *BMI* Body mass index, *DH* Disc herniation, *DS* Degenerative spondylolisthesis, *FS* Foraminal stenosis, *PLF* Posterolateral fusion, *PLIF* Posterior lumbar interbody fusion, *BMD* Bone mineral density

### Relationship between age and ASD based on radiological outcomes

Radiologic measurements of the preexisting spinal stenosis and disc degeneration at the adjacent segments showed that the degree of preoperative LDD did not significantly differ between the ASD and N-ASD groups (Table 2). Among the preoperative spinal parameters, the PI-LL was larger in the ASD group compared with the N-ASD group (16.58 ± 13.22 vs 7.37 ± 11.06, *p* <  0.001). After surgery, the ASD group had a significantly smaller LL (38.14 ± 13.81 vs 44.98 ± 9.41, *p* = 0.002) and larger PI-LL (16.38 ± 13.10 vs 6.28 ± 14.19, *p* <  0.001) than the N-ASD group. The △LL was also larger in the ASD group than the N-ASD group (12.18 ± 6.79 vs 7.74 ± 5.06, *p* <  0.001) (Table 2). The correlation between the PI-LL angle and △LL was analyzed. In patients > 60 years of age, when both PI-LL and △LL were divided into three groups (cut-off value of 10°and 20°), the differences between the ASD and N-ASD groups were statistically significant (Table 3). When PI-LL was divided into two groups, the cut-off value of 20°(*p* = 0.008) instead of 10°(*p* = 0.083) was statistically significant. When △LL was divided into two groups, the cut-off value of 10°(p = 0.008) instead of 20°(*p* = 0.231) was statistically significant. However, in patients ≤60 years of age, a high prevalence of ASD was significantly associated with PI-LL ≥ 10° (*p* = 0.013), and △LL was not associated with ASD.

**Table 2:** Univariate analysis comparing radiographic variables between patients with and without adjacent segment disease

Characteristic	ASDis (n = 62)	N-ASD (n = 62)	χ^2^/t	*p*
LL (°)				
Preoperative	37.94 ± 13.11	44.00 ± 8.74	−3.02	0.003
Postoperative	38.14 ± 13.81	44.98 ± 9.41	−3.23	0.002
SS (°)				
Preoperative	36.37 ± 10.35	34.18 ± 7.99	1.32	0.189
Postoperative	33.41 ± 10.13	31.81 ± 9.10	0.923	0.358
PT (°)				
Preoperative	18.15 ± 11.64	17.19 ± 10.37	0.485	0.629
Postoperative	21.12 ± 10.32	19.46 ± 9.26	0.941	0.348
PI (°)	54.52 ± 10.45	51.37 ± 12.06	1.56	0.122
PI-LL (°)				
Preoperative	16.58 ± 13.22	7.37 ± 11.06	4.21	< 0.001
Postoperative	16.38 ± 13.10	6.28 ± 14.19	4.12	< 0.001
△LL (°)	12.18 ± 6.79	7.74 ± 5.06	4.13	< 0.001
Preexisting spinal stenosis at adjacent segment				
Yes	32 (51.6%)	24 (38.7%)	2.08	0.149
No	30 (48.4%)	38 (61.3%)		
Preexisting disc degeneration at adjacent segment				
Yes	24 (38.7%)	16 (25.8%)	2.36	0.124
No	38 (61.3%)	46 (74.2%)		

△LL = |Postoperative LL - Preoperative LL|

**Table 3:** Comparing correlation of postoperative pelvic parameter between patients with and without adjacent segment disease in different age groups

Pelvic parameter	Age ≤ 60			Age > 60		
	ASDis (*n* = 28)	N-ASD (n = 28)	P	ASDis (*n* = 34)	N-ASD (n = 34)	P
LL (°)	39.45 ± 9.31	45.06 ± 11.17	0.046	37.06 ± 16.71	44.92 ± 7.84	0.016
SS (°)	37.90 ± 9.10	34.41 ± 7.76	0.419	35.27 ± 10.87	30.80 ± 9.53	0.076
PT (°)	22.61 ± 12.47	18.70 ± 9.36	0.190	19.88 ± 8.14	20.08 ± 9.27	0.926
PI (°)	53.77 ± 9.89	51.74 ± 11.69	0.488	55.15 ± 10.99	50.87 ± 12.60	0.141
PI-LL (°)						
< 10	6 (21.4%)	15 (53.6%)	0.045	10 (29.4%)	17 (50%)	0.025
10–20	16 (57.1%)	9 (32.1%)		12 (35.3%)	14 (41.2%)	
> 20	6 (21.5%)	4 (14.3%)		12 (35.3%)	3 (8.8%)	
△LL (°)						
< 10	16 (57.1%)	20 (71.4%)	0.497	12 (35.3%)	23 (67.6%)	0.027
10–20	8 (28.6%)	6 (21.4%)		17 (50%)	9 (26.5%)	
> 20	4 (14.3%)	2 (7.1%)		5 (14.7%)	2 (5.9%)	

### Logistic regression analysis

**Fig. 2:**
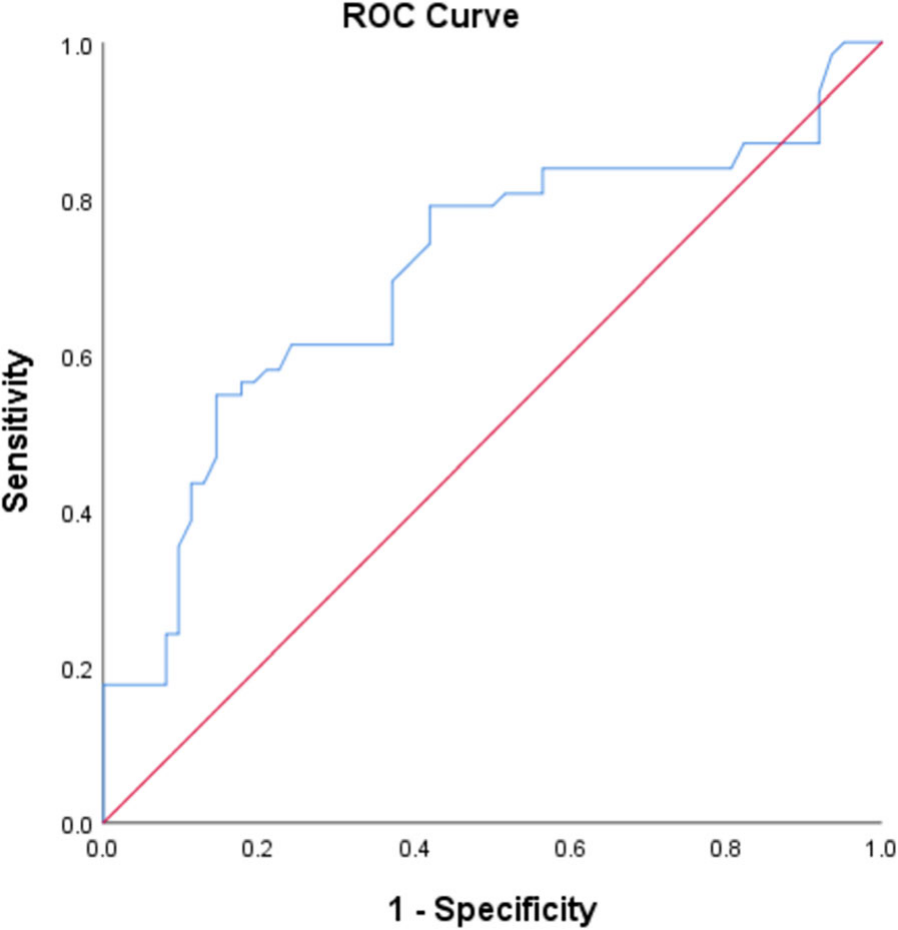
Logistic regression and receiver operating characteristic curve analysis show a cut-off value for postoperative change in lumbar lordosis (△LL) of 11.7° at which the classification based on △LL yields a sensitivity of 58% and specificity of 86%. The area under the curve is 0.703, with a confidence interval of 0.608 to 0.798

Multivariate logistic regression analysis was performed to determine the relative impact of radiographic features on the incidence of ASD. After adjusting for the variables age, BMI, sex, PI-LL, △LL, surgical level, number of fused segments, preexisting disc degeneration, and preexisting spinal stenosis at the adjacent segment, the variables that were associated with the development of ASD were a small postoperative LL angle (OR = 0.96, *p* = 0.023), PI-LL > 10° (OR = 2.72, *p* = 0.014), and △LL > 12° (OR = 3.11, *p* = 0.007) (Table 4). The receiver operating characteristic curve analysis for measurements of △LL revealed that a cutoff value of 11.7° was able to distinguish between the two groups with the highest sensitivity and specificity, with an area under the curve of 0.703 (95% confidence interval 0.608–0.798) (Fig. 2).

**Table 4:** Result from Univariate and multivariate logistic regression analysis for potential risk factors for ASDis

	Univariate		Multivariate	
Variables	OR (95%CI)	*p*	OR (95%CI)	*p*
Lumbar BMD (T scores)	0.84 (0.64–1.10)	0.2	–	–
Postoperative LL (°)	0.95 (0.92–0.99)	0.005	0.96 (0.92–0.99)	0.023
Postoperative SS (°)	1.02 (0.98–1.06)	0.355	–	–
Postoperative PT (°)	1.02 (0.98–1.06)	0.346	–	–
Postoperative PI (°)	1.03 (0.99–1.06)	0.114	–	–
Postoperative PI-LL (°)				
< 10	Reference			
> 10	2.82 (1.34–5.97)	0.007	2.72 (1.22–6.04)	0.014
△LL(°)				
< 12	Reference			
> 12	3.43 (1.58–7.45)	0.002	3.11 (1.37–7.08)	0.007
Preexisting disc degeneration at adjacent segment	1.82 (0.85–3.90)	0.126	–	–
Preexisting spinal stenosis at adjacent segment	1.69 (0.83–3.45)	0.15	–	–
Segments fused	1.14 (0.56–2.31)	0.718	–	–
Fusion method	1.39 (0.68–2.84)	0.365	–	–

## Discussion

The maintenance of spinopelvic alignment is most important for adults with spinal deformity, as this is the primary determinant of life quality after corrective surgery [15]. However, sagittal imbalance reportedly increases the probability of ASD after spinal fusion for LDD [7]. LL is important for maintaining sagittal balance and upright posture. The most widely-used method of measuring LL is to measure the Cobb angle between the upper endplate of L1 and the upper endplate of S1 in the standing position. Currently, the relationships between LL and age, sex, and other factors are unclear; however, LL is positively correlated with lumbar spondylolisthesis and spondylolysis, and negatively correlated with LBP [10, 15]. Failure to maintain normal LL may also increase the incidence of facet arthritis [16]. If the LL is small, this increases the risk of sagittal imbalance after surgery and is a predictor of ASD [17], which is similar to our findings. Thus, restoration of the physiological curvature of the lumbar spine is very important in improving patient quality of life and preventing postoperative complications.

The spinopelvic balance plays an important role in LDD. Several formulas have been created to evaluate the ideal LL to be reestablished in lumbar fusion surgery in different populations. Based on Legaye’s formula in Korean patients [18], Lee et al. [9] found that overcorrection of LL (postoperative LL angle > ideal LL) effectively maintains the optimal SVA in patients with degenerative lumbar kyphosis during a minimum 2-year follow-up. Considering the effect of age, Xu et al. [19] determined the predictive formula for the ideal LL in Chinese adults as: LL = 0.508 × PI - 0.088 × age + 28.6. Therefore, the surgical reconstruction of the ideal LL must consider variables such as age and ethnicity. Given that the normal range of LL varies widely (18.5–72.3° using the Cobb method) [20], it is difficult to estimate the normal/optimal LL angle for an individual.

**Fig. 3:**
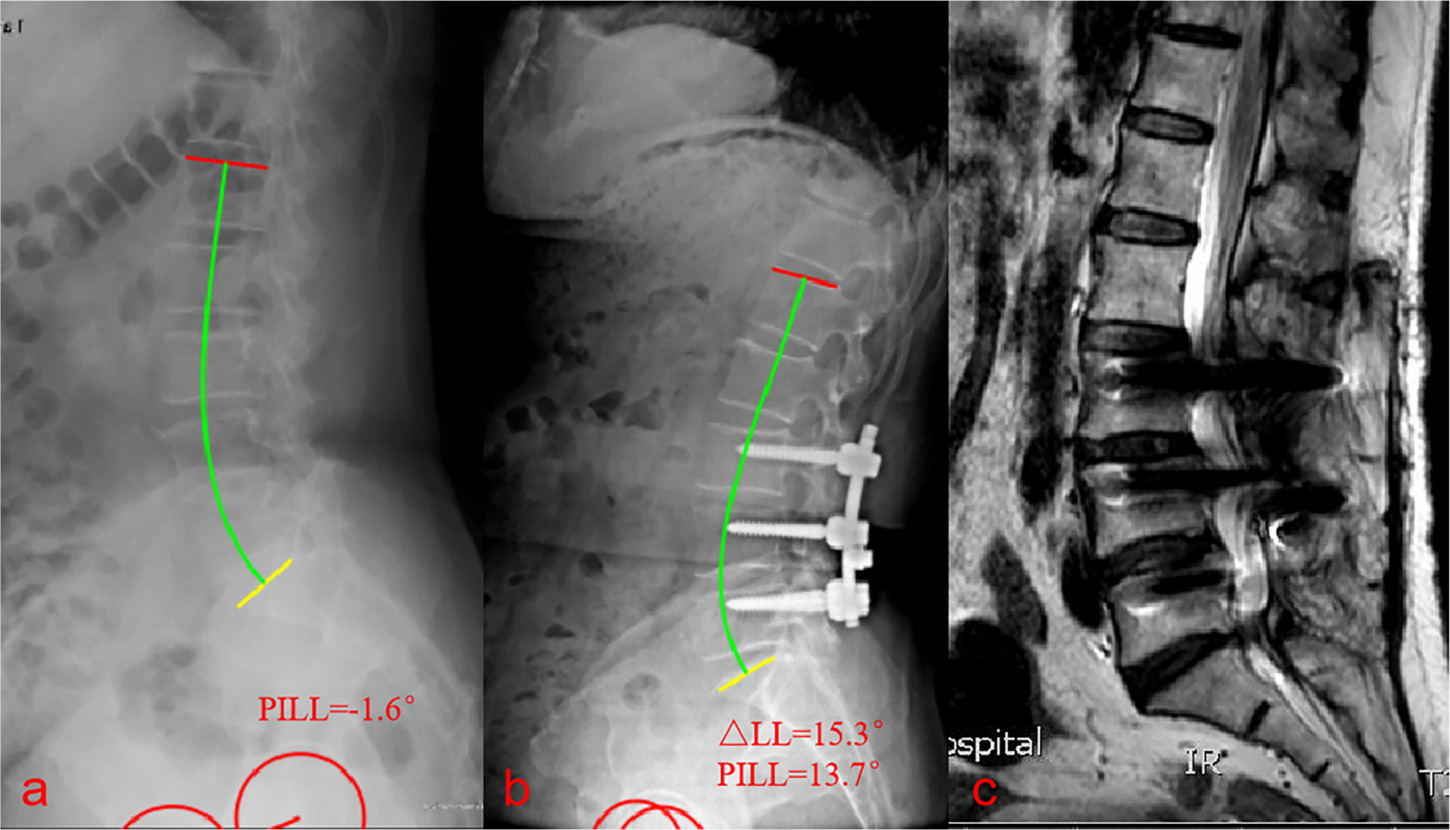
Images from a 65-year-old man who underwent three-segment spinal fusion. **a** Preoperative radiograph. **b** 1-week postoperative radiograph. **c** MRI revealing the development of symptomatic adjacent segment disease at 3 years and 5 months postoperatively. Preoperatively, the pelvic incidence to lumbar lordosis mismatch (PI-LL) was − 1.6°. Postoperatively, the PI-LL was 13.7° and the change in lumbar lordosis was 15.3°. Patients (age > 60) with a change in lumbar lordosis of > 10° were significantly more likely to develop adjacent segment disease

There is not enough existing knowledge to accurately reconstruct the lordotic curvature. Our study attempted to explore the relationship between the △LL and the need for reoperation for ASD after lumbar fusion in patients of different ages. A △LL of > 10° was associated with an increased risk of ASD in patients > 60 years old (Fig. 3), but not in patients ≤60 years old. This suggests that surgeons should not markedly change the LL angle in older adults. No previous study has investigated the effect of the postoperative change in LL on the prevention of ASD. However, the regional Cobb angle of L4-S1 is reportedly a crucial factor affecting the formation of LL [20], and a review of the data from 274 patients found that a postoperative L4–S1/L1–S1 lordosis ratio of < 50% increased the prevalence of ASD [21]. Further studies are required to confirm the ideal correction of the L4–S1/L1–S1 lordosis ratio and △LL.

**Fig. 4:**
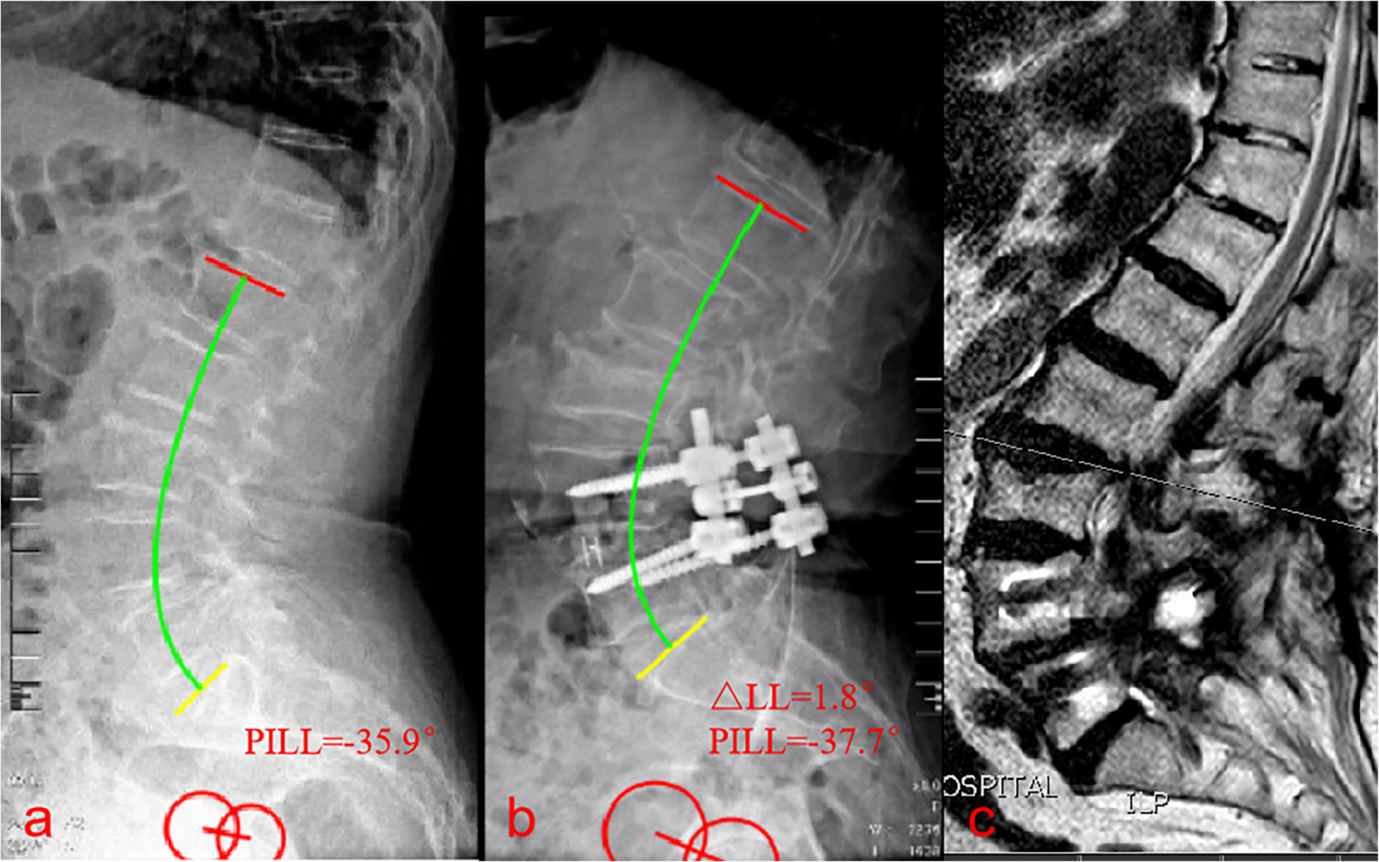
Images from a 71-year-old woman who underwent one-segment spinal fusion. **a** Preoperative radiograph. **b** 1-week postoperative radiograph. **c** MRI revealing the development of symptomatic adjacent segment disease at 1 year and 3 months postoperatively. Preoperatively, the pelvic incidence to lumbar lordosis mismatch (PI-LL) was − 35.9°. Postoperatively, the PI-LL was − 37.7° and the change in lumbar lordosis was 1.8°. Patients (age > 60) with a PI-LL of > 20° were significantly more likely to develop adjacent segment disease

**Fig. 5:**
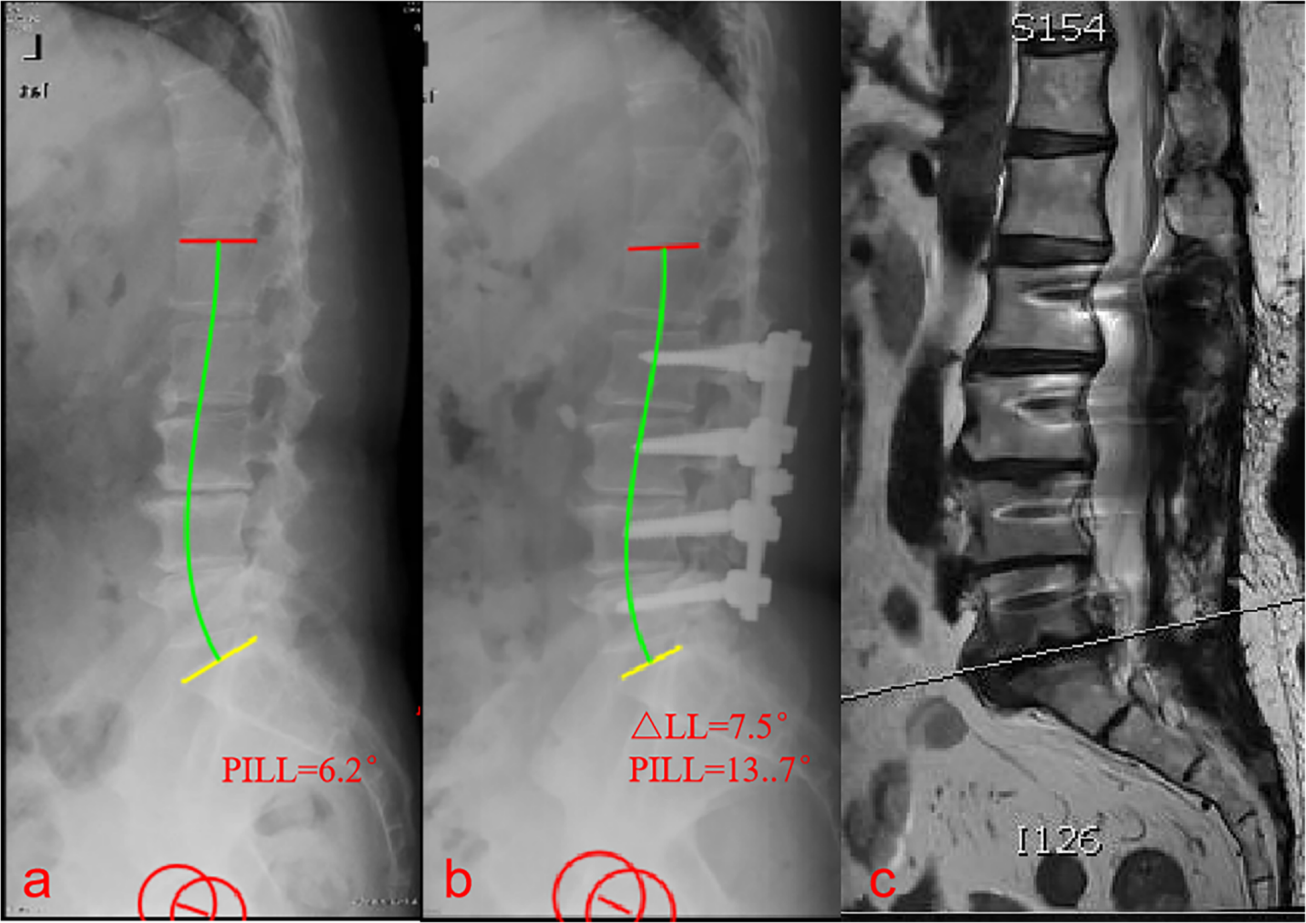
Images from a 58-year-old man who underwent four-segment spinal fusion. **a** Preoperative radiograph. **b** 1-week postoperative radiograph. **c** MRI revealing the development of symptomatic adjacent segment disease at 2 years and 1 month postoperatively. Preoperatively, the pelvic incidence to lumbar lordosis mismatch (PI-LL) was 6.2°. Postoperatively, the PI-LL was 13.7° and the change in lumbar lordosis was 7.5°. Patients (age ≤ 60) with a PI-LL of > 10° were significantly more likely to develop adjacent segment disease

**Fig. 6:**
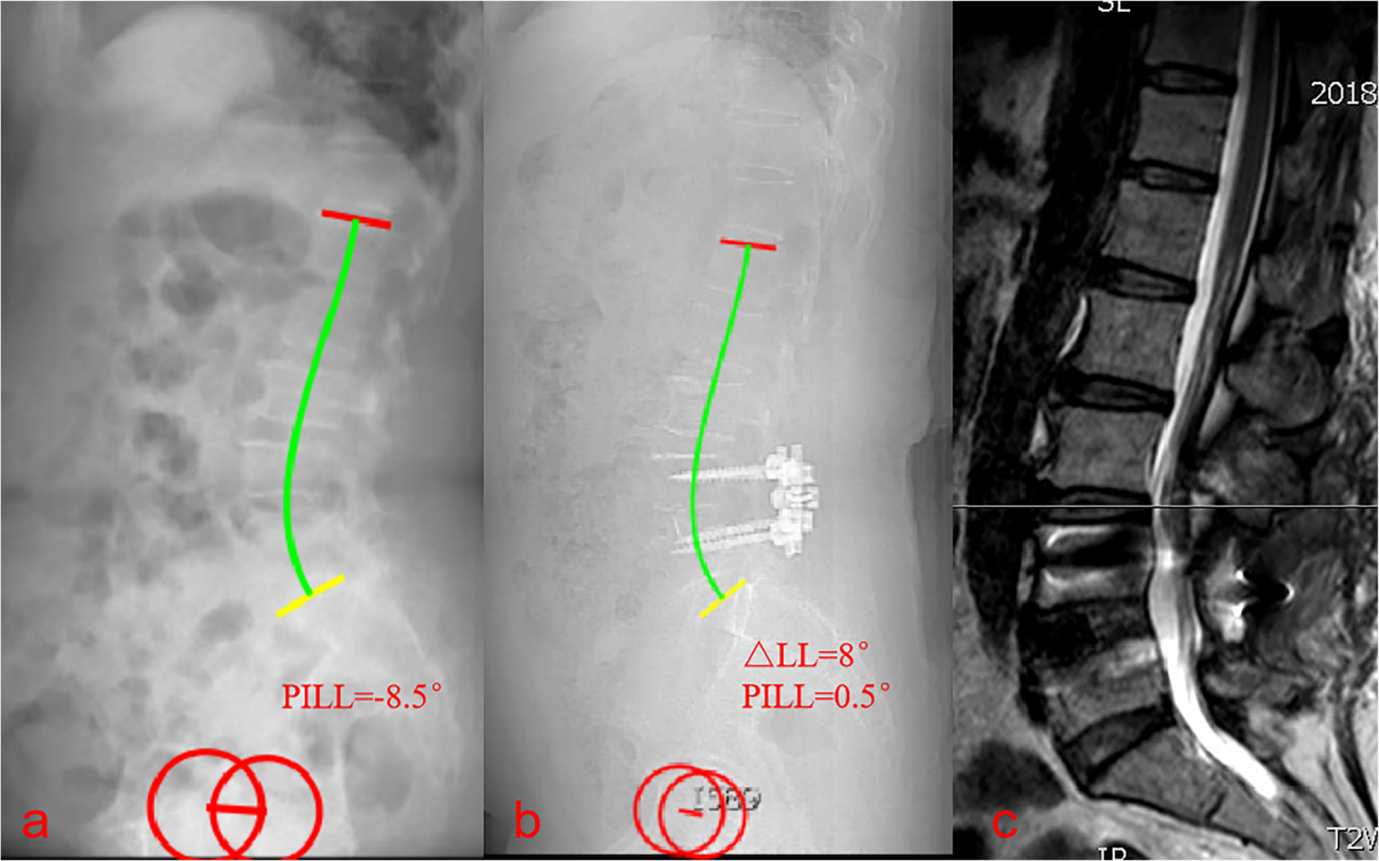
Images from a 56-year-old woman who underwent one-segment spinal fusion. **a** Preoperative radiograph. **b** 1-week postoperative radiograph. **c** MRI revealing the development of symptomatic adjacent segment disease at 4 years and 6 months postoperatively. Preoperatively, the pelvic incidence to lumbar lordosis mismatch (PI-LL) was − 8.5°. Postoperatively, the PI-LL was 0.5° and the change in lumbar lordosis was 8°. Patients with a PI–LL of < 10° and a change in lumbar lordosis of < 10° were significantly less likely to develop adjacent segment disease

A recent study reported that the variables most related to severe disability (Oswestry Disability Index > 40) due to adult spinal deformity are a PT of > 22 °, SVA of > 47 mm, and PI-LL of > 11° [22]. Based on age-specific Oswestry Disability Index values, a subsequent study revealed that the ideal spinopelvic alignment values for patients aged < 35 years are a PT of 10.9°, PI-LL of 10.5°, and SVA of 4.1 mm, while those for patients aged > 75 years are a PT of 28.5°, PI-LL of 16.7°, and SVA of 78.1 mm [12]. PI-LL mismatch can also be used to predict the incidence of ASD after spinal fusion surgery. Rothenfluh et al. [11] reported that patients with a PI-LL of ≥10° were 10 times more likely to undergo revision surgery than those with a PI-LL of < 10°. Sagittal imbalance after lumbar fusion may increase the incidences of postoperative complications and ASD. In the present study, patients > 60 years old with a PI-LL of > 20° had an increased incidence of ASD (Fig. 4). However, a PI-LL of > 10° was associated with a high prevalence of ASD in patients ≤60 years old (Fig. 5). Patients with a PI-LL of ≥10° experience greater shear stresses and compression forces at the intervertebral joints after lumbar fusion compared with those with a PI-LL of < 10°, which may indicate a poor natural history [5]. Figure 6 highlights a patient with a PI–LL of < 10° and a change in lumbar lordosis of < 10° were significantly less likely to develop adjacent segment disease.

For every adult, the PI is fixed and is a reliable morphological parameter of the human body. The size of the PI-LL mismatch reveals the relative decrease in LL, resulting in the displacement of the gravity axis of the PI and the inhomogeneity of the sagittal alignment of the spine [23]. When the sagittal plane of the spine is unbalanced, the body will instigate a series of compensatory mechanisms to maintain the balance of the sagittal plane. The first compensatory mechanism of the spine is overextension of the thoracic vertebrae, which reduces the thoracic kyphosis [24]. The later compensation tends to manifest as retrodisplacement and posterior translation of the pelvis, along with flexion of the knees and ankles [25]. Clinically, the trunk of older adults is pitched forward due to loss of LL, and so they can withstand degenerative sagittal imbalances. Thus, it may be counterproductive to fully return the spinal curvature to normal in older adults. Our current strategy is to determine the appropriate LL and PI-LL at the time of surgery to prevent ASD via long-term or short-term fusion. To obtain the optimal LL and PI-LL, surgeons should consider using methods such as appropriate hyper wedge cages and the bend screw-rod system that can meet normal physiological curve of the spine.

The present study had some limitations. (1) The data were obtained from cases of spinal surgery performed in a single institution. (2) The relationship between LL and quality of life was not assessed. However, as the assessment was based only on radiological measurements, the data were relatively objective. (3) The optimal LL angle varies in accordance with ethnicity, age, sex, and other variables. Our study cohort only represents a demographically homogenous group of Chinese patients.

## Conclusion

The occurrence of symptomatic ASD after spinal fusion is strongly associated with a smaller LL angle, greater PI-LL mismatch, and excessive △LL. The LL required to prevent symptomatic ASD in older adults differs from that in younger adults, as the ideal correction of LL varies with increasing age. Therefore, these factors should be considered and a corresponding surgical strategy should be selected to reduce the risk of reoperation for ASD.

## Data Availability

The datasets used and analyzed during the current study are available from the corresponding author on reasonable request.

## Abbreviations

ASDis: Adjacent segment disease
ASD: Adjacent segment degeneration
LL: Lumbar lordosis
PI: Pelvic incidence
SS: Sacral slope
PT: Pelvic tilt
SVA: Sagittal vertical axis
△LL: Postoperative LL - preoperative LL
LDD: Lumbar degenerative disease
LBP: Low back pain
PLF: Posterolateral fusion
PLIF: Posterior lumbar interbody fusion
BMI: Body mass index
MRI: Magnetic resonance imaging
PSM: Propensity score-matched
